# Preoperative amygdala fMRI in temporal lobe epilepsy

**DOI:** 10.1111/j.1528-1167.2008.01739.x

**Published:** 2009-02

**Authors:** Silvia B Bonelli, Robert Powell, Mahinda Yogarajah, Pamela J Thompson, Mark R Symms, Matthias J Koepp, John S Duncan

**Affiliations:** NSE MRI Unit, Dept of Clinical and Experimental Epilepsy, Institute of Neurology UCLQueen Square, London, United Kingdom

**Keywords:** Temporal lobe epilepsy, fMRI, Emotion, Amygdala.

## Abstract

**Purpose:**

Anterior temporal lobe resections (ATLR) benefit 70% of patients with refractory mesial temporal lobe epilepsy (TLE), but may be complicated by emotional disturbances. We used functional magnetic resonance imaging (fMRI) to investigate the role of the amygdala in processing emotions in TLE and whether this may be a potential preoperative predictive marker for emotional disturbances following surgery.

**Methods:**

We studied 54 patients with refractory mesial TLE due to hippocampal sclerosis (28 right, 26 left) and 21 healthy controls using a memory encoding fMRI paradigm, which included viewing fearful and neutral faces. Twenty-one TLE patients (10 left, 11 right) subsequently underwent ATLR. Anxiety and depression were assessed preoperatively and 4 months postoperatively using the Hospital Anxiety and Depression Scale.

**Results:**

On viewing fearful faces, healthy controls demonstrated left lateralized, while right TLE patients showed bilateral amygdala activation. Left TLE patients had significantly reduced activation in left and right amygdalae compared to controls and right TLE patients. In right TLE patients, left and right amygdala activation was significantly related to preoperative anxiety and depression levels, and preoperative right amygdala activation correlated significantly with postoperative change of anxiety and depression scores, characterized by greater increases in anxiety and depression in patients with greater preoperative activation. No such correlations were seen for left TLE patients.

**Discussion:**

The fearful face fMRI paradigm is a reliable method for visualizing amygdala activation in controls and patients with mesial TLE. Activation of the right amygdala preoperatively was predictive of emotional disturbances following right ATLR.

Temporal lobe epilepsy (TLE) and anterior temporal lobe resection (ATLR) are associated with both cognitive and emotional disturbances. The amygdala is involved in emotional and social behavior, fear conditioning, face perception, and facial expression processing (Cahill et al., 1995; Morris et al., 1996, 1998a). In humans, amygdala lesions can lead to deficits in the recognition of, especially, fearful facial expression (Morris et al., 1996) and impaired fear conditioning (LaBar et al., 1995).

In patients with TLE, mesial temporal sclerosis affects the hippocampus, the entorhinal cortex, and the amygdala complex (Williamson et al., 1993), which may underlie emotional symptoms and changes that are present before and develop following ATLR. Isolated amygdala damage is observed in 10% of patients with TLE (Bartlett et al., 2002) and often accompanies hippocampal sclerosis (HS) (Van Paesschen et al., 1996). Functional magnetic resonance imaging (fMRI) has been used to study memory function in patients with refractory TLE and HS. It has been suggested that hippocampal and parahippocampal activation may help the prediction of postoperative neuropsychological deficits (Rabin et al., 2004; Richardson et al., 2004b, 2006; Janszky et al., 2005).

Few studies have used fMRI paradigms capable of lateralizing amygdala activation during presurgical assessment of patients with TLE (Richardson et al., 2004a; Schacher et al., 2006), and the utility as a clinical tool remains uncertain. An animated fearful face paradigm resulted in bilateral amygdala activation in healthy volunteers, while in patients with mesial TLE, amygdala activation was clearly lateralized to the contralateral side (Schacher et al., 2006). Amygdala-hippocampal codependence was shown during emotional-memory encoding. Severity of amygdala pathology was a predictive factor for memory for emotional items, and severity of hippocampal pathology predicted memory performance for neutral and emotional items (Richardson et al., 2004a).

Several psychiatric studies have shown atypical amygdala responsiveness, mostly hyperactivity, being associated with anxiety disorders (Thomas et al., 2001; Killgore & Yurgelun-Todd, 2005) and major depressive disorder (Abercrombie et al., 1998; Roberson-Nay et al., 2006).

Between 20% to 50% of the patients with TLE suffer from comorbid psychiatric symptoms, most commonly anxiety and depression (Ring et al., 1998; Kanner, 2003; Boylan et al., 2004). Up to 50% of patients with no psychiatric history may develop symptoms of anxiety and depression shortly after ATLR (Blumer et al., 1998; Ring et al., 1998), but postoperative emotional disturbances have received less attention than cognitive changes, and the underlying mechanisms are poorly understood. So far, only a few prognostic indicators for emotional disturbances after ATLR (preoperative anxiety and depression, right ATLR, postoperative surgical outcome, structural MRI changes) have been described. The aim of this study was to: (1) Investigate the role of the amygdala in processing emotions in healthy controls as well as in patients with left and right TLE and (2) test the hypotheses that, in patients with TLE, stronger fMRI activation in the amygdala would be associated with higher anxiety and depression ratings, and greater amygdala fMRI activations preoperatively would be associated with greater risk of mood disturbances after ATLR. If confirmed, preoperative amygdala fMRI might then be used as an additional preoperative predictor for mood disturbances following ATLR.

## Patients and Methods

### Subjects

We studied 54 consecutive patients with medically refractory TLE due to unilateral HS [26 left (15 female); median age, 42; range, 18–62 years; 28 right (12 female); median age, 37; range, 21–52 years]. All patients were undergoing presurgical evaluation at the National Hospital for Neurology and Neurosurgery, London, and had undergone structural MRI at 3T, including qualitative assessment by expert neuroradiologists and quantification of hippocampal volumes and T2 relaxation times (Woermann et al., 1998; Bartlett et al., 2007). Hippocampal volumetry demonstrated a normal contralateral hippocampus in all subjects. Video electroencephalography (EEG) confirmed seizures arising from the ipsilateral mesial temporal lobe in all 54 patients. All patients underwent language- and memory-fMRI and standard neuropsychological assessment preoperatively and 4 months postoperatively.

All patients' first language was English. Handedness was determined using a standardized questionnaire (Oldfield, 1971); language dominance was assessed using a range of fMRI tasks (Powell et al., 2006), revealing left hemisphere dominance in 39 patients, bilateral language representation in 10 patients, and 5 patients with atypical, right hemisphere dominance. Left TLE patients had on average 2.33 simple partial seizures (SPS), 6.77 complex partial seizures (CPS), and 0.21 generalized tonic–clonic (GTC) seizures per month; right TLE patients had 5.85 SPS, 8.38 CPS, and 0.65 GTC on average per month. All patients were treated with antiepileptic drugs, without changes made to medication between the preoperative fMRI scan and the 4-month postoperative follow-up. To date, 10 of 26 left and 11 of 28 right TLE patients have had an ATLR. There was no difference in the size of the temporal lobe resection between the left and right TLE groups.

We also studied 21 right-handed native English-speaking healthy volunteers (median age 47, range 22–62 years; 10 female) with no history of neurological and psychiatric disease. Twenty controls were right-handed, one was left-handed, and all were left language dominant as assessed by the fMRI language tasks.

The study was approved by the National Hospital for Neurology and Neurosurgery and the Institute of Neurology Joint Research Ethics Committee, and written informed consent was obtained from all subjects.

### Anxiety and depression scale

The Hospital Anxiety and Depression Scale (HADS) (Zigmond & Snaith, 1983) was used as a measure of self**-**reported symptoms of anxiety and depression. The scale is a user-friendly, compact questionnaire comprising 14 items that assess current levels of anxiety and depression. The score is derived from responses on a four-point Likert-type scale. A score of 7 or above is considered positive. Patients completed this scale before and 4 months after ATLR. In those patients who underwent ATLR, measures of anxiety and depression change following surgery were calculated as postoperative-preoperative scores. Changes in anxiety and depression scores from baseline following ATLR were then correlated with preoperative fMRI activation patterns. A clinically significant change was defined by a change in category; the different categories are defined as follows: normal (0–6), mild (7–10), moderate (11–13), severe (14 and above).

### MR data acquisition

MRI studies were performed on a 3T General Electric Excite HD scanner. Standard imaging gradients with a maximum strength of 40 mTm^−1^ and slew rate 150 Tm^−1^s^−1^ were used. All data were acquired using an eight-channel array head coil for reception and the body coil for transmission. For each subject, we acquired a high-resolution echo planar image (EPI) covering the whole brain.

For the fMRI task, gradient-echo planar T^*_2_^-weighted images were acquired, providing blood oxygenation level-dependent (BOLD) contrast. Each volume comprised 44 contiguous 1.5-mm oblique axial slices through the temporal and frontal lobes, with a 24-cm field of view, 128 × 128 matrix, and in-plane resolution of 1.88 × 1.88 mm. Echo time (TE) was 30 ms, and repetition time (TR) was 4.5 s. The field of view was positioned to cover the temporal lobe with the anterior-posterior axis aligned with the long axis of the hippocampus on sagittal views and with the body of the hippocampus in the center. The imaging time series was realigned, normalized into standard anatomical space using the high-resolution whole brain EPI and smoothed with a Gaussian kernel of 10 mm full-width half maximum.

### fMRI paradigm

Stimuli of three different material types [pictures (P), words (W), and faces (F)] were visually presented to the subjects during a single scanning session. This paradigm was used to investigate verbal and nonverbal memory encoding as described previously (Powell et al., 2007). In brief, a total of 210 stimuli were presented, one every 4 s, in seven cycles. Each cycle consisted of a block of 10 pictures (black and white nameable line drawn objects), 10 words (single concrete nouns), and 10 faces (partly black and white, partly colored photographs unfamiliar to the subjects), followed by 20 s of crosshair fixation. During scanning, subjects were explicitly instructed to perform a deep encoding task which involved making a judgement on whether each stimulus was pleasant or unpleasant. This task was used in order to encourage stimulus encoding, but was not used in any subsequent parts of the fMRI analysis. The faces used consisted of 23 fearful, 23 happy, and 24 neutral faces. Each block of faces contained a balanced mixture of the three expression types.

### Data analysis

Imaging data were analyzed with Statistical Parametric Mapping (SPM2) (Friston et al., 1995). A two-level event-related random-effects analysis was used.

At the first level, for each subject, trial-specific responses were modeled by convolving a delta function that indicated each event onset with the canonical hemodynamic response function (HRF) to create regressors of interest, one regressor for each of the three event types (neutral, fearful, and happy faces). Each subject's movement parameters were included as confounds, and parameter estimates pertaining to the height of the HRF for each regressor of interest were calculated for each voxel. One contrast image for the main effect of viewing fearful compared to neutral faces and one for the main effect of viewing happy compared to neutral faces was created for each subject. These images were then used for the second-level analysis.

At the second level of the random effects analysis, we divided the subjects into three groups: Healthy volunteers, left TLE, and right TLE patients. Within each group, each subject’s contrast images were entered into a one-sample *t*-test to examine the main effects of viewing fearful and happy faces compared with neutral faces. Two-sample *t*-tests were performed to highlight brain regions demonstrating more or less activation in one group compared to another.

In order to test for correlations between areas of fMRI activation and the subject’s performance on the anxiety and depression questionnaires pre- and postoperatively, multiple regression analyses were performed for each voxel over the whole brain. For each subject, we used the anxiety and depression scores as covariates for fMRI activation during the fearful face paradigm. Furthermore, the measures of change of anxiety and depression scores were used to test for correlations between preoperative fMRI activation and change in anxiety and depression scores from before to 4 months after ATLR in those patients who had had ATLR and postoperative neuropsychological assessment.

We report all medial temporal lobe (MTL) activations at a threshold of p < 0.001, uncorrected for multiple comparisons, if not stated otherwise. This uncorrected threshold was adopted because of the low signal-to-noise ratio in the anterior temporal lobe and as we were testing a specific hypothesis regarding MTL activation. MTL regions of activation were labeled with reference to Duvernoy’s The Human Hippocampus (Duvernoy, 1998).

## Results

### Hospital anxiety and depression scale—scores

Preoperatively, there was no significant difference in anxiety and depression scores between left and right TLE patients (anxiety—median: left TLE, 7; range, 1–18; right TLE, 9; range, 3–15; depression—median: left TLE, 5.5; range, 0–10; right TLE, 4.5; range, 0–15). The median scores in our controls' data were 5.5 for anxiety (range, 0–13) and 1.5 for depression (range, 0–5).

Five out of 10 patients undergoing left ATLR had a postoperative decline in anxiety scores, three had an increase in anxiety scores, and two remained unchanged. The mean change between pre- and postoperative anxiety score was −0.5 (range, −8 to +3). Seven left TLE patients had a postoperative decline in depression scores, one an increase, and two remained unchanged. The mean change between pre- and postoperative depression score was −1.5 (range, −6 to +1).

Four out of 11 patients undergoing right ATLR had a postoperative decline in anxiety scores, six had an increase, and one patient's score remained unchanged. The mean change between pre- and postoperative anxiety score was +1.0 (range, −5 to +7). Four right TLE patients had a postoperative decline in depression scores, six had an increase, and one patient remained unchanged. The mean change between pre- and postoperative depression score was +1.0 (range, −7 to +6).

A clinically significant change in anxiety was observed in four out of 10 left TLE patients (three patients with an improvement, one with worsening of anxiety) and a clinically significant change in depression in two out of 10 left TLE patients (two patients with an improvement in depression).

In right TLE patients, we found a clinically significant change in anxiety in seven out of 11 patients (three patients with an improvement and four with worsening of anxiety) and a clinically significant change in depression in four out of 11 patients (two patients with an improvement and two with worsening of depression).

There were no statistically significant correlations between preoperative anxiety or depression ratings and postoperative change in anxiety or depression.

Postoperative outcome was classified according to the International League Against Epilepsy (ILAE) classification proposed by Wieser and coworkers (2001). There was no significant correlation between postoperative outcome and change in anxiety or depression scores at 4 months and therefore postoperative seizure control was not a factor in changes of anxiety or depression after ATLR.

### Hippocampal volumes and amygdala T2 maps

Left and right hippocampal volumes were significantly different in both left and right TLE patients; left TLE group: mean (SD) right hippocampal volume, 2.79 (0.30) cm^3^; mean left hippocampal volume, 1.81 (0.46) cm^3^ (paired *t*-test p < 0.0001, 2-tailed); right TLE group: mean right hippocampal volume, 2.00 (0.58) cm^3^; mean left hippocampal volume, 2.62 (0.35) cm^3^ (paired *t*-test p < 0.0001, 2-tailed). There was no significant difference between left hippocampal volume in the left TLE group and right hippocampal volume in the right TLE group. There was no significant difference of amygdala T_2_ values between right and left TLE patients within each group.

### fMRI activations

We report all significant activations within the amygdala, the hippocampus, and the parahippocampal structures for the contrast of viewing fearful compared to neutral faces.

Activation peaks in the MTL for the main effects and their interactions across each group are given in Table 1a and Table 1b.

**Table 1b:** Two sample t-tests showing two way interactions between patients and controls for the main effects of viewing fearful faces contrasted by neutral faces in the medial temporal lobe

		Uncorrected	Coordinates (x, y, z)	
Subjects	Z-score	p-value	in MNI space	Anatomical region
Left TLE < controls	3.75	0.000	50, −20, −2	Right superior temporal gyrus
	3.63	0.000	22, −8, −12	Right hippocampus/amygdala
	3.17	0.001	32, −4, −28	Right amygdala
	3.22	0.001	−26, −8, −8	Left insula
	3.29	0.000	−20, −10, −20	Left amygdala
	2.90	0.002	52, −4, −6	Right insula
Left TLE > controls	ns	ns	—	—
Right TLE < controls	3.38	0.001	48, −22, −2	Right superior temporal gyrus
	2.28-ns	0.011-ns	−16, 0, −14	Left amygdala
	1.69-ns	0.025-ns	20, −2, −14	Right amygdala
Right TLE > controls	ns	ns	—	—
Left TLE > right TLE	ns	ns	—	—
Right TLE > left TLE	3.77	0.000	32, −4, −26	Right amygdala
	3.48	0.000	10, −12, −20	Right hippocampus
	3.39	0.000	22, −8, −16	Right amygdala/hippocampus
	3.67	0.000	−18, −12, −20	Left hippocampus
	3.40	0.000	−28, −6, −28	Left amygdala

TLE, temporal lobe epilepsy; ns, not significant.

**Table 1a:** One-sample *t*-test showing fMRI activation peaks for the main effects of viewing fearful faces contrasted by neutral faces in the MTL across groups (TH 0.001)

		Uncorrected	Coordinates (x, y, z)	
Subjects	Z-score	p-value	in MNI space	Anatomical region
Controls	2.80	0.003	−24, 2, −26	Left amygdala
	2.31	0.011	14, 2, −22	Right amygdala
	4.07	0.000	50, −20, −2	Right superior temporal gyrus
	3.09	0.001	52, −40, −18	Right fusiform gyrus
	3.09	0.001	−52, −20, −10	Left superior temporal gyrus
	3.10	0.001	−40, −46, −22	Left fusiform gyrus
Left TLE patients	ns	ns	—	—
Right TLE patients	3.21	0.001	−24, 2, −30	Left amygdala
	3.13	0.001	26, 0, −26	Right amygdala

MNI space, coordinates related to a standard brain defined by the Montreal Neurological Institute (MNI); TLE, temporal lobe epilepsy; ns, not significant.

#### Controls

**Figure 1:**
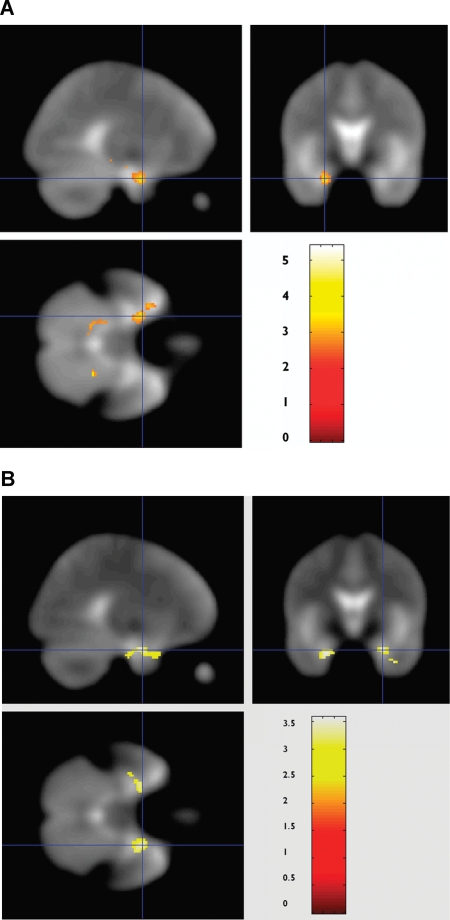
(**A** and **B**) Main effect of viewing fearful contrasted by neutral faces in controls (**A**) left amygdala activation, and in patients with right temporal lobe epilepsy (**B**) bilateral amygdala activation. Color scale indicating increasing *t*-values.

There was left-sided amygdala activation on viewing fearful faces (p = 0.003) (Fig. 1A). Activation was also seen in the right amygdala; however, this did not reach statistical significance (p = 0.011).

#### Right TLE

Both, left and right amygdala (p = 0.001) activations were observed on viewing fearful faces (Fig. 1B).

#### Left TLE

No significant MTL activation was seen for viewing fearful faces.

### Group comparisons

For group comparisons, see Table 1b.

#### Left TLE versus controls

Left TLE patients activated significantly less in the left (p = 0.000) and right amygdala (p = 0.001) compared to controls. Left TLE patients also demonstrated significantly less activation than controls in the right hippocampus (p = 0.000).

#### Right TLE versus controls

Right TLE patients showed less activation in the left (p = 0.011, uncorrected) and right (p = 0.025, uncorrected) amygdala than controls; however, this did not reach statistical significance.

#### Left TLE versus right TLE

**Figure 2:**
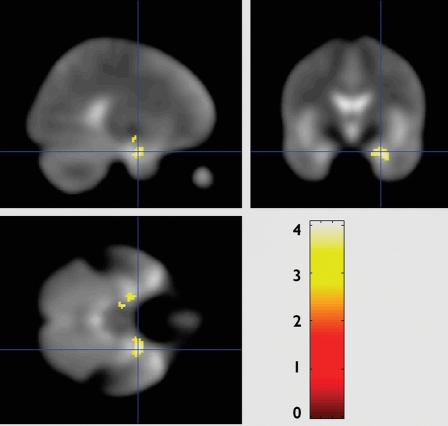
Significantly more fMRI activation on viewing fearful contrasted by neutral faces in both, left and right amygdala in patients with right temporal lobe epilepsy compared to patients with left temporal lobe epilepsy.

Compared to right TLE patients, left TLE patients activated significantly less in the left and right amygdala (p = 0.000) and left and right hippocampus (p = 0.000) (Fig. 2).

For the contrast “happy compared to neutral faces,” we did not find any significant activations within the temporal lobe at the group levels. Therefore, we did not perform correlations between this contrast and scores of anxiety and depression.

#### Correlational analysis

**Figure 3:**
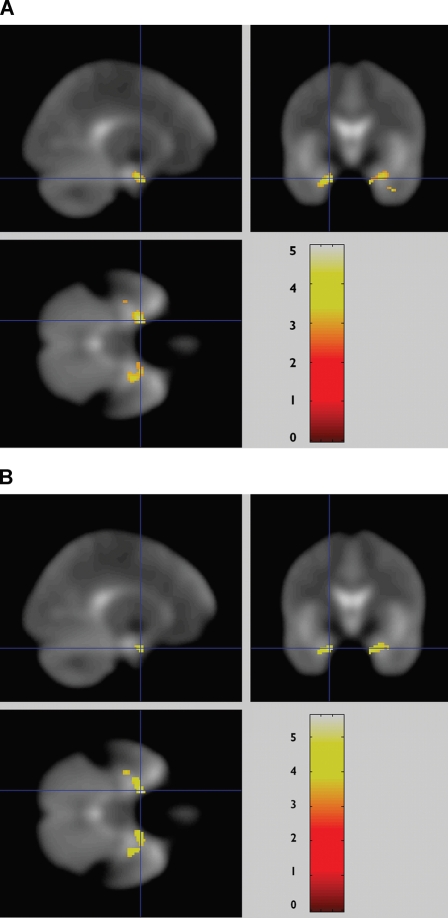
(**A** and **B**) Right TLE group. Multiple regression analysis calculated for each voxel over the whole brain. Positive correlation of preoperative fMRI activation on viewing fearful contrasted by neutral faces and preoperative anxiety (**A**) or preoperative depression (**B**) scores in left and right amygdale.

Correlation of fMRI activation (viewing fearful contrasted by neutral faces) with preoperative anxiety/depression scores: In right TLE patients, there was a significant correlation between preoperative anxiety scores and fMRI activation associated with viewing fearful faces in left (p = 0.001) and right amygdala (p = 0.000) (Fig. 3A and Table 1c). Right TLE patients also showed a significant correlation between preoperative depression scores and fMRI activation associated with viewing fearful faces in left and right amygdala (p = 0.000) (Fig. 3B). No significant correlations were demonstrated for left TLE patients or healthy controls.

**Table 1c:** Correlations of preoperative fMRI activation on viewing fearful faces contrasted by neutral faces and preoperative anxiety and depression scores over the whole brain

			Uncorrected	Coordinates (x y z)	
	Subjects	Z-score	p-value	in MNI space	Anatomical region
Correlation of preoperative fMRI activation	Controls	ns	ns	—	—
versus preoperative anxiety scores	Left TLE	ns	ns	—	—
	Right TLE	4.00	0.001	−20, 0, −26	Left amygdala
		3.29	0.000	30, −4, −24	Right amygdala
		3.17	0.001	16, 0, −28	Right amygdala
Correlation of preoperative fMRI activation	Controls	ns	ns	—	—
versus preoperative depression scores	Left TLE	ns	ns	—	—
	Right TLE	4.34	0.000	−20, 0, −26	Left amygdala
		3.95	0.000	16, 0, −28	Right amygdala

TLE, temporal lobe epilepsy; ns, not significant.

**Figure 4:**
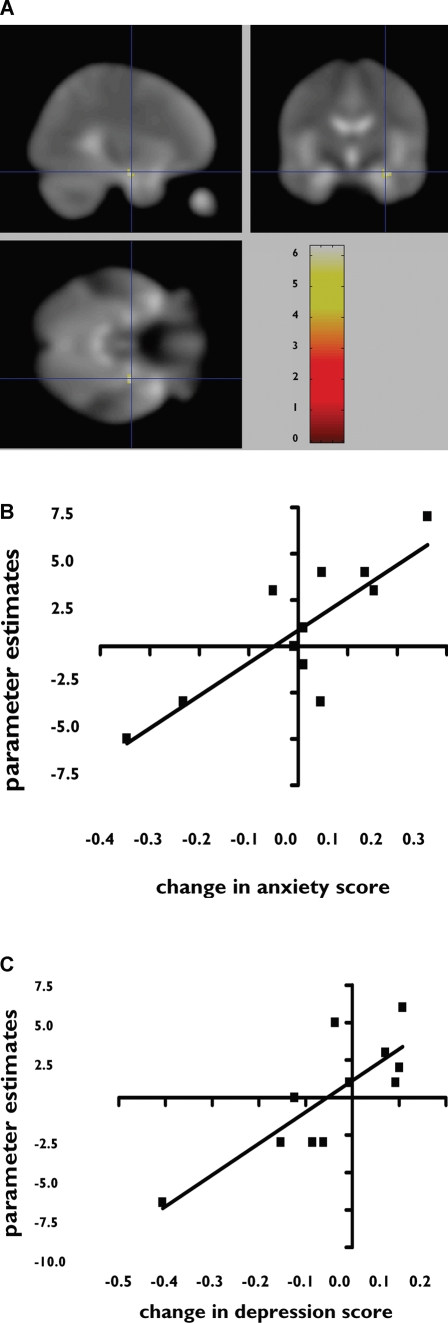
(**A** and **B**) Right TLE group. Multiple regression analysis—parameter estimates calculated for each voxel over the whole brain. Preoperative right amygdala activation with fearful contrasted by neutral faces correlates with postoperative change in anxiety scores, characterized by a greater increase in anxiety scores in patients with greater preoperative activation. (**C**) Right TLE group. Multiple regression analysis—parameter estimates calculated for each voxel over the whole brain. Preoperative right amygdala activation with fearful contrasted by neutral faces correlates with postoperative change in depression scores, characterized by a greater increase in depression scores in patients with greater preoperative activation.

Correlation of fMRI activation (viewing fearful contrasted by neutral faces) with change in anxiety/depression scores postoperatively: 21 patients (11 right and 10 left TLE) underwent ATLR and completed mood ratings 4 months following surgery (Table 1d). In right TLE patients, there was a significant correlation between fMRI activation and postoperative change in anxiety scores in the right amygdala (p = 0.000), characterized by a greater increase in anxiety scores in patients with greater preoperative fMRI activation (Fig. 4, A and B). In right TLE patients, there was also a significantly positive correlation between fMRI activation and postoperative change in depression scores in the right amygdala (p = 0.000), characterized by a greater increase in depression scores in patients with greater preoperative fMRI activation (Fig. 4C). In left TLE patients, we did not observe any significant correlation between preoperative fMRI activation on viewing fearful faces and postoperative change in anxiety or depression scores.

**Table 1d:** Correlations of preoperative fMRI activation on viewing fearful contrasted by neutral faces and postoperative change in anxiety and depression scores over the whole brain

				Coordinates	
			Uncorrected	(x, y, z)	
	Subjects	Z-score	p-value	in MNI space	Anatomical region
Correlation of preoperative fMRI	Right TLE (11 subjects)	3.91	0.000	30, −8, −20	Right amygdala
activation versus postoperative	Left TLE (10 subjects)	ns	ns	—	—
change in anxiety scores					
Correlation of preoperative fMRI	Right TLE	3.37	0.000	20, −2, −10	Right amygdala
activation versus postoperative		3.20	0.001	18, 2, −10	Right amygdala
change in depression scores		4.09	0.000	−66, −30, −12	Left middle temporal gyrus
		3.98	0.000	52, −10, −20	Right middle temporal gyrus
	Left TLE	ns	ns	—	—

TLE, temporal lobe epilepsy; ns, not significant.

## Discussion

We demonstrated the feasibility of amygdala fMRI and compared the role of the amygdala in processing emotion in healthy controls and in patients with medically refractory TLE using a fearful face paradigm. Furthermore, we demonstrated that in these patients, atypical amygdala responsiveness was significantly related to pre- and postoperative levels of anxiety and depression.

The paradigm proved to be easy to perform due to its low cognitive demands and allowed evaluation of hippocampal and amygdala functional integrity in a single session. Controls demonstrated a left lateralized pattern of amygdala activation, while patients showed reduced amygdala activation compared to controls.

Significant correlations were seen in the right TLE patients between amygdala activation and levels of preoperative anxiety and depression, characterized by higher preoperative fMRI activation correlating with higher anxiety and depression scores. Finally, those right TLE patients who had undergone surgery also demonstrated correlations between preoperative right amygdala activation on viewing fearful faces and postoperative change in anxiety and depression levels, with greater preoperative activation being associated with worsening of anxiety and depression scores following ATLR.

Our findings corroborate previous studies showing asymmetric activations in response to viewing fearful stimuli in normal subjects and extend these findings to patients with refractory epilepsy. Using positron emission tomography (PET), Morris et al. (1996) observed a significantly greater neural response to fearful as opposed to happy faces in the left amygdala and therefore provided evidence of a differential neural response to facial expressions of fear and happiness. Schacher et al. (2006) reported symmetric bilateral amygdala activation on presentation of fearful faces in controls, whereas 12 patients with either right or left mesial TLE had an activation pattern that was lateralized to the contralateral side. A possible explanation for apparent discrepancy between the current findings and those of Schacher in 2006 might be that, in contrast to our experiment, the paradigm used in the latter study combined the dynamic perception of motion with the presentation of fearful facial expressions to maximize signal change mainly within the amygdalae, while we used static stimuli. In patients, the severity and extent of the underlying pathology may also contribute to the functional dissociation in the various patient groups as amygdala and hippocampal sclerosis may be combined, but the sclerotic process may be restricted to the hippocampus or the amygdala. A recent cortical stimulation study suggested a functional lateralization of the amygdalae for different types of stimuli. Electrical stimulation of the right amygdala induced negative emotions, especially fear and sadness, whereas stimulation of the left amygdala led to either pleasant (happiness) or unpleasant (fear, anxiety, sadness) emotions (Lanteaume et al., 2007). Other studies have suggested a specific role for the right amygdala in automatic stimuli processing, while more detailed decoding of the importance of stimuli might be associated with activity of the left amygdala (Morris et al., 1998b; Wright et al., 2003). This functional dissociation might be a reason for the differing results in our left TLE and right TLE patients. Left TLE patients demonstrated less left amygdala activation, most likely due to pathology. We also observed less activation in the right amygdala, possibly because left and right amygdalae do not function as independently as has been suggested by the “functional dissociation hypothesis” (Morris et al., 1998b; Lanteaume et al., 2007).

Medically refractory TLE is associated with an increased prevalence of emotional disorders, in particular mood and anxiety disorders (Gilliam et al., 2003; Boylan et al., 2004), which may be related to dysfunction of the limbic system, in particular the amygdala and the hippocampus. In patients with TLE, the amygdala is often affected by sclerosis, together with the hippocampus (Williamson et al., 1993; Van Paesschen et al., 1996). The hippocampus is critical for memory encoding, and hippocampus and amygdalae are responsible for emotional memory encoding (Richardson et al., 2004a). As the amygdala is one of the key structures for emotional processing (Morris et al., 1996; Morris et al., 1998a) and social perception, surgical trauma to a normal amygdala may result not only in impairment of emotional memory (Cahill et al., 1995) but also in emotional and social disturbances (Adolphs, 2003).

In the current study, we found a correlation between preoperative bilateral amygdala activation and anxiety and depression levels preoperatively in right TLE patients, characterized by greater anxiety and depression scores with greater fMRI activation. In contrast, left TLE patients did not show a correlation between amygdala activation and anxiety and depression scores. This suggests that the right amygdala has a key role in feelings of anxiety and depression, and that dysfunction in this structure is associated with increased anxiety and depression, the degree of which is associated with increased activation.

Our results are in keeping with psychiatric studies in which a positive correlation was found between amygdala activation and measures of anxiety and depression using fMRI as a predictor for treatment outcome in anxiety disorders (McClure et al., 2007). Amygdala involvement has also been linked with fear auras at the onset of seizures (Cendes et al., 1994) and with aggressive outbursts in patients with TLE (van Elst et al., 2000). Abnormally high resting activity of the amygdala has been described in patients with major depression (Abercrombie et al., 1998).

Several studies have evaluated potential predictive factors for postoperative psychiatric disorders, such as preoperative depression or anxiety disorder (Quigg et al., 2003; Devinsky et al., 2005), right ATLR (Quigg et al., 2003), and postoperative seizure control (Devinsky et al., 2005), but prediction of postoperative psychiatric symptoms remains a challenge. Irrespective of seizure outcome, patients are at risk of either developing de novo mood disorders or continuing to suffer from anxiety or depression after ATLR (Blumer, 2002; Blumer et al., 2002).

We observed a clinically significant improvement in depression only in two out of 10 patients with left TLE and two out of 11 patients with right TLE following surgery, while only two patients with right TLE significantly deteriorated.

In three out of 10 left TLE and three out of 11 right TLE patients we found a clinically significant improvement in anxiety after surgery, while one left TLE patient and four right TLE patients significantly worsened. The analysis used the actual change scores. Reducing the data to increased/decreased scores would not be appropriate for a study with this number of subjects and would result in loss of sensitivity.

We found a positive correlation in right TLE patients between preoperative right amygdala activation on viewing fearful faces and postoperative increase in anxiety and depression scores. As with previous studies, there was no relation between seizure outcome and mood disturbances after ATLR (Wrench et al., 2004). We conclude from our findings that resection of a right amygdala that is reacting strongly to emotional stimuli, albeit less than controls, results in increased risk of emotional disturbances, at least at 4 months following ATLR. The inference is that a dysfunctional right amygdala has a modulating role on emotion and mood. There are, of course, no data to determine the effects of removal of a normal right amygdala on anxiety and depression.

Occasionally, a significant improvement of preexisting mood disturbances after epilepsy surgery is reported (Spencer et al., 2003; Reuber et al., 2004; Devinsky et al., 2005). Results are contradictory with regard to lateralizty. Some studies suggested a higher risk of (de novo) psychiatric symptoms after right ATLR (Glosser et al., 2000; Quigg et al., 2003), others for left ATLR (Piazzini et al., 2001), while others found no effect of laterality (Devinsky et al., 2005).

Multiple mechanisms are likely to contribute to the evolution of postoperative mood symptoms. The higher rate of anxiety and depression in TLE patients in the early postoperative stage suggests that neurobiological mechanisms play a part (Kanner & Balabanov, 2002; Krishnamoorthy et al., 2002). Disruption, removal, or deafferentation of the limbic system structure after ATLR is most obvious. In the longer term, psychosocial factors such as the “burden of normality” are likely to be contributory (Wilson et al., 2001).

There are several methodological limitations of the study. First, our study focused on activations within the MTL, and therefore our imaging parameters were not optimal for studying neocortical regions outside the temporal lobe. We cannot, therefore, report on other extratemporal/limbic systems that are likely to be involved in emotional processing, such as the orbitofrontal regions.

Secondly, our postoperative results are based on a follow-up of only 4 months, with a relatively small sample of 21 patients. Several studies have shown that there is a temporal pattern to psychiatric morbidity after surgery with up to 50% of the patients developing psychiatric symptoms within 6 weeks, but symptoms of depression tend to improve or resolve by 1 year follow-up, which seems less true for comorbid anxiety disorders (Ring et al., 1998).

Third, the HADS is a scale of self-reported symptoms of anxiety and depression. The results, based on these scores, are preliminary and merit further evaluation using more detailed diagnostic instruments.

In conclusion, fMRI with emotional stimuli activates the amygdalae in controls and to a lesser extent in those with right TLE and even less in left TLE. The correlation of amygdala activation with anxiety and depression in those with right TLE suggests a modulatory role for the right amygdala on mood. Of potential clinical relevance, the degree of activation of the right amygdala in those with right TLE was predictive of postoperative mood disturbances, and this may be a useful tool to identify those at risk of this morbidity.
